# Anticancer activity of paroxetine in human colon cancer cells: Involvement of MET and ERBB3

**DOI:** 10.1111/jcmm.14011

**Published:** 2018-11-13

**Authors:** Won‐Jun Jang, Sung Keun Jung, Tam Thuy Lu Vo, Chul‐Ho Jeong

**Affiliations:** ^1^ College of Pharmacy Keimyung University Daegu Korea; ^2^ School of Food Science and Biotechnology Kyungpook National University Daegu Korea

**Keywords:** apoptosis, colon cancer, MET, paroxetine, SSRI

## Abstract

The concept of drug repositioning has recently received considerable attention in the field of oncology. In the present study, we propose that paroxetine can be used as a potent anticancer drug. Paroxetine, one of the selective serotonin reuptake inhibitors (SSRIs), has been widely prescribed for the treatment of depression and anxiety disorders. Recently, SSRIs have been reported to have anticancer activity in various types of cancer cells; however, the underlying mechanisms of their action are not yet known. In this study, we investigated the potential anticancer effect of paroxetine in human colorectal cancer cells, HCT116 and HT‐29. Treatment with paroxetine reduced cell viability, which was associated with marked increase in apoptosis, in both the cell lines. Also, paroxetine effectively inhibited colony formation and 3D spheroid formation. We speculated that the mode of action of paroxetine might be through the inhibition of two major receptor tyrosine kinases – MET and ERBB3 – leading to the suppression of AKT, ERK and p38 activation and induction of JNK and caspase‐3 pathways. Moreover, in vivo experiments revealed that treatment of athymic nude mice bearing HT‐29 cells with paroxetine remarkably suppressed tumour growth. In conclusion, paroxetine is a potential therapeutic option for patients with colorectal cancer.

## 1 INTRODUCTION

Many cancers, including colorectal cancer (CRC), are still difficult to treat. CRC is the third most common cancer in both men and women, and was ranked second responsible for cancer‐related deaths globally in 2015.^1^ Approximately 30% of CRC patients in Europe and the United States have metastasis at the time of diagnosis,^1^, ^2^ suggesting that metastasis of CRC is mainly responsible for the global mortality burden. Metastatic CRC has been treated with 5‐fluorouracil, which has remained the first choice of chemotherapy drug for many years, and several new targeted drugs such as humanized monoclonal antibody against EGFR (cetuximab) or anti‐VEGFR (bevacizumab) have been recently used for the treatment of CRC patients.^3^ However, the newly introduced drugs mostly showed very modest benefits at high costs and were often related to drug resistance. Therefore, the development of efficient drugs by exploiting new pathways and mechanisms of CRC metastasis is necessary.

Drug repositioning, the identification of new therapeutic indications for applying already approved drugs for other diseases, is recently gaining considerable attention.^4^ A major advantage of this approach is that extensive clinical data on how a drug works in the body and its potential toxicity are already available. Furthermore, it might be a very effective or alternative strategy to develop anticancer drugs, because the conventional anticancer drug development process is particularly long and expensive. Thus, drug repositioning has a wide scope in oncology as a promising approach for the development of new treatments.^5^ This approach has been widely applied to determine the first‐line treatments for rare tumours, for which effective treatments are lacking, and to develop second‐line regimens for relapsed disease.^6^ Recently, some nononcological drugs have been repurposed as anticancer drugs, and related clinical trials are ongoing. For example, thalidomide was approved for the treatment of multiple myeloma in 2016. In addition, several lines of evidence also support the active recruiting clinical trials involving repurposed drugs in various tumour types, such as thalidomide, disulfiram, digoxin, aspirin, and metformin.^6^

Antidepressants are widely used to treat depression and other mood disorders.^7^, ^8^ Several classes of antidepressants, including tricyclic antidepressants (TCAs), serotonin‐norepinephrine reuptake inhibitors, and selective serotonin‐reuptake inhibitors (SSRIs), are available. Recent studies have shown that certain TCAs and SSRIs exhibit potent anticancer activities as well as psychotropic effects.^9^, ^10^ In a previous epidemiological study, daily intake of SSRI was shown to reduce the risk of CRC, whereas no protective effect was found for TCAs.^11^ In addition to other drugs belonging to SSRIs (eg, fluoxetine and sertraline), paroxetine is a potent SSRI, which has proven to be effective in treating generalized anxiety and major depressive disorders.^12^ Moreover, paroxetine has been reported to have immunosuppressive effects in human lymphocytes 13 and exerts marked cytotoxic effect in several types of cancer cells.^14^ In a previous study, SSRIs such as sertraline and paroxetine were shown to exhibit pro‐apoptotic activity in the human CRC cell line, HT29.^15^ However, the authors also provided conflicting in vivo data that sertraline, but not paroxetine, inhibited tumour growth in HT29‐xenograft nude mice^15^; further studies are needed to confirm this. Therefore, in this study, we aimed to evaluate the anticancer activity of paroxetine in human CRC cells and in vivo xenograft nude mice and suggest novel molecular mechanisms underlying its apoptotic effect.

## 2 MATERIALS AND METHODS

### 2.1 Materials

Paroxetine HCl, an anti‐depressant agent, was obtained from APExBIO (Houston, TX, USA). Antibodies for cleaved caspase‐8, cleaved caspase‐3, PARP, p‐EGFR (Tyr1068), p‐ERBB3 (Tyr1289 and Tyr1328), ERBB3, p‐ERBB2 (Tyr1248), ERBB2, p‐MET (Tyr1234/1235), MET, p‐AXL (Tyr702), AXL, p‐IGF‐1Rb (Tyr1131), IGF‐1Rb, p‐AKT (Ser437), AKT, p‐ERK (Thr202/Tyr204), ERK, p‐p38 (Thr108/Tyr182), p38, p‐JNK (Thr183/Tyr185), and JNK were purchased from Cell Signaling Technology (Beverly, MA, USA). Antibodies specific for Bcl‐2, GAPDH, and EGFR and the secondary antibody were purchased from Santa Cruz Biotechnology (Santa Cruz, CA, USA). Halt protease and phosphatase inhibitor cocktail (100×), EDTA (100×), and bicinchoninic acid (BCA) protein assay kit were obtained from Thermo Fisher Scientific (Rockford, IL, USA). MTT and DMSO were obtained from Sigma‐Aldrich (St. Louis, MO, USA). PVDF membranes were purchased from Bio‐Rad (Hercules, CA, USA). SuperSignal West Dura Extended Duration Substrate was purchased from Thermo Scientific (Waltham, MA, USA). A human phospho‐RTK array kit was purchased from R&D Systems (Minneapolis, MN, USA).

### 2.2 Cell culture

The human HCT116 and HT29 colon adenocarcinoma cell lines were maintained in RPMI‐1640 medium containing l‐glutamine supplemented with 10% (v/v) heat‐inactivated FBS, 100 U/mL penicillin, and 0.1 mg/mL streptomycin under 5% CO_2_ at 37°C.

### 2.3 Cell viability assay

Cell viability was measured using the MTT assay. HCT116 and HT29 cells were distributed at 2.5 × 10^3^ cells/well in 96‐well microplates. After 24 hours of incubation, the cells were treated with different concentrations of paroxetine and incubated for 24 and 48 hours in a 37°C incubator. Subsequently, 20 μL of MTT (5 mg/mL) was added to each well and incubated for 4 hours. The medium was removed from the wells, and 200 μL DMSO was added to each well and agitated for 3 minutes. Absorbance at 570 nm was detected using FLUOster Omega (BMG Labtech).

### 2.4 3D spheroid culture

HCT116 and HT29 cells were suspended and counted. 2.5 ×10^3^ cells were dispensed into each well of the ultra‐low attachment surface‐coated spheroid microplate (Corning, Tewksbury, MA, USA) in the presence of various concentrations of paroxetine. For 3D spheroid formation, normal growth RPMI 1640 medium supplemented with 10% FBS was used, and the cells were maintained at 37°C in a 5% CO_2_ incubator for 1 week. The spheroid images were captured at various time‐points by using a light microscope. The size of spheroids was determined using ImageJ software.

### 2.5 Phospho‐RTK array

Phospho‐RTK array analysis was performed using a human phospho‐RTK array kit (R&D Systems), according to the product manual. Briefly, HCT116 and HT29 cells were seeded on 100 mm culture dishes at 2 × 10^6^ cells. The cells were treated with 10 μmol/L of paroxetine and incubated for 24 hours. The cell lysates were prepared using NP40‐lysis buffer containing the protease‐phosphatase inhibitor cocktail, PMSF, and EDTA. After the arrays were blocked for 1 hour with Array Buffer 1, they were incubated with 400 μg of protein lysates overnight at 4°C. The arrays were then washed and incubated with an HRP‐conjugated phospho‑tyrosine detection antibody. The arrays were detected by chemiluminescence and imaged using LAS‐3000, according to manufacturer's instructions. The intensity of the average signal of the pair of duplicated spots was calculated relative to that of the negative control spots.

### 2.6 Western blot analysis

HCT116 and HT29 cells were seeded on 100 mm culture dishes at 2 × 10^6^ cells and incubated for 24 hours. The cells were treated with the indicated concentrations of paroxetine for 24 hours. The cells were harvested in cold‐NP40 lysis buffer (50 mmol/L Tris‐HCl pH 8.0, 150 mmol/L NaCl, 1% NP‐40) supplemented with Halt™ Protease and phosphatase inhibitor cocktail, PMSF, and EDTA. The cells were lysed on ice for 30 minutes and centrifuged at 16 000 *g* for 30 minutes at 4°C. The concentration of total proteins was quantified using the BCA protein assay. Next, 30 μL of protein was separated using SDS‐PAGE and transferred to PVDF membranes. The membranes were blocked with 5% BSA in TBS plus 0.1% Tween (TBS‐T) at room temperature for 2 hours and then incubated with the specific primary antibodies overnight at 4°C. After the membranes were washed with 0.1% TBS‐T 3 times for 15 minutes each, they were incubated with the HRP‐conjugated secondary antibody at room temperature for 1 hour. Proteins were visualized using the SuperSignal West Dura Extended Duration Substrate. The images were analysed using LAS‐3000 (Fuji, Japan) according to manufacturer's instructions.

### 2.7 Annexin V apoptosis analyses

Apoptosis was detected using the annexin V‐FITC apoptosis detection kit, as recommended by the manufacturer (MBL international Corp., Watertown, MA). Cells were treated with vehicle and paroxetine for 24 hours, fixed in 70% ethanol, and stored at −20°C for 24 hours. After the cells were stained with annexin V, apoptosis was determined using a BD FACS Calibur Flow Cytometer (BD Biosciences, San Jose, CA).

### 2.8 Xenograft assay

Male athymic nude mice (5 weeks old; mean body weight, 20 g) were obtained from Orient (Seoul, South Korea). Animals were acclimated for 1 week before the study and maintained under specific pathogen‐free conditions based on the guidelines established by the Seoul National University Animal Care and Use Committee. HT‐29 cells (2 × 10^6^ cells/100 μL) were suspended in RPMI‐1640 medium and subcutaneously inoculated with 100 μL matrigel into the left flank of each mouse. When tumours reached a size of 100 mm^3^, mice were divided into three groups: (a) vehicle group (n =* *8); (b) 1 mg/kg paroxetine (n* *=* *8); and (c) 5 mg/kg paroxetine (n* *=* *8). Vehicle and paroxetine were injected intraperitoneally three times per week for 2 weeks. Tumour size was measured three times per week by using calipers, and tumour weight was recorded after excision on the day of the termination of the experiment. Tumour volume was calculated according to a standard formula: tumour volume (mm^3^) =(length × width × height × 0.5). Mice were monitored until the tumours reached 1 cm^3^ in total volume and were killed for further studies.

### 2.9 Statistics

Quantitative data are shown as the mean value ± SD unless indicated otherwise. The statistical significance of compared values was analysed using two‐tailed Student's *t* test or one‐way ANOVA followed by Bonferroni test. All statistical analyses were performed using GraphPad Prism software. *P* values <0.05 were considered statistically significant.

## 3 RESULTS

### 3.1 Paroxetine suppresses the growth of CRC cells

**Figure 1:**
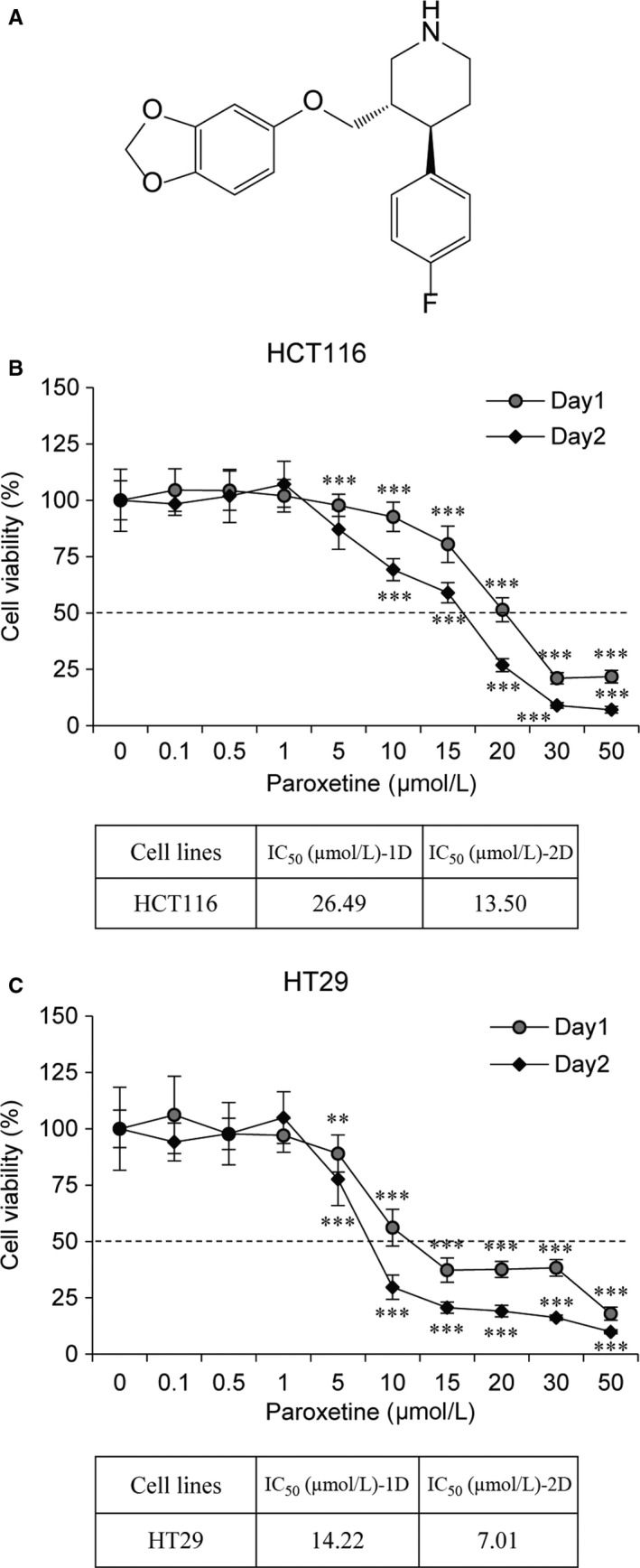
The effects of paroxetine on cell viability in HCT116 and HT29 cells. A, Chemical structure of paroxetine. (B‐C) Viability of paroxetine‐treated HCT116 and HT29 cells. HCT116 and HT29 cells were seeded onto 96‐well plates (1 × 10^3^ cells/well) and treated with various concentrations of paroxetine for 48 h. Cell viability was measured using the MTT assay. Data are shown as the mean ± SD (n = 4). Statistical analysis was conducted using one‐way ANOVA followed by Bonferroni test. **P <* *0.01; ***P <* *0.001 vs untreated control. IC_50_ was determined using nonlinear regression by using GraphPad Prism software

Recent studies have shown that SSRIs are able to reduce the growth and survival of various cancer cells.^16^, ^17^, ^18^, ^19^ The anti‐growth effect of paroxetine (Figure 1A) on human CRC cells was assessed by treating HCT116 and HT29 cells with different concentrations of paroxetine for 2 days, and cell viability was determined using the MTT assay. Data revealed that treatment with paroxetine decreased cell viability in a dose‐dependent manner in both HCT116 and HT29 cells. The half maximal (50%) inhibitory concentration (IC_50_) values for paroxetine were found to be 26.49 μmol/L (Day1) and 13.50 μmol/L (Day2) in HCT116 cells or 14.22 μmol/L (Day1) and 7.01 μmol/L (Day2) in HT29 cells, respectively (Figure 1B, C). Interestingly, HT29 cells were more sensitive to paroxetine than HCT116 cells.

### 3.2 Paroxetine inhibits anchorage‐independent colony and spheroid formation of HCT116 and HT29 cells

**Figure 2:**
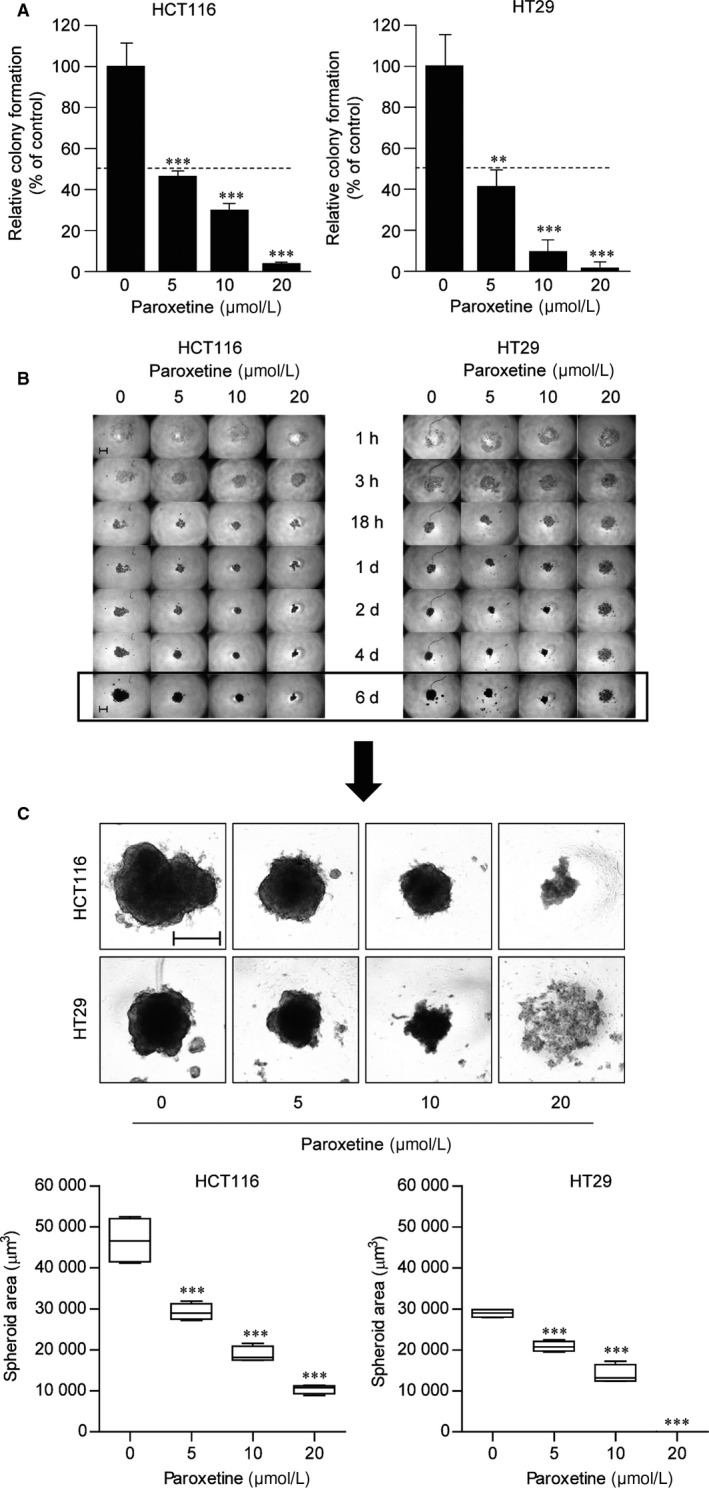
Anti‐proliferative activity of paroxetine in colony and spheroid formation of HCT116 and HT29 cells. A, Anchorage‐independent growth of HCT116 and HT29 cells. Cells were seeded onto 6‐well soft agar plates (8 × 10^3^ cells/well) and incubated for 6 d. Colony images were obtained using a light microscope. Random areas in colonies grown in soft agar were scanned (five areas per well, three wells per set). Error bars represent mean ± SD (n* *=* *15). Statistical significance was determined using a Student's *t* test (**P <* *0.01, ***P <* *0.001). B, The effects of paroxetine on the growth of HCT116 and HT29 spheroids. Cells (2.5 × 10^3^ cells/well) were dispensed into each well of an ultra‐low attachment surface‐coated 96‐well plate. The cells were treated with various concentrations of paroxetine and incubated for 6 d. The spheroid images were obtained using a light microscope. C, Optical images and box‐ and whisker plots of HT29 and HCT116 cell spheroids; scale bar: 200 μm. Spheroid area was determined using Image J software. Statistical analysis was conducted using one‐way ANOVA with Bonferroni test. ***P <* *0.001, compared to untreated group

The anticancer effect of paroxetine on HCT116 and HT29 cells was investigated by performing anchorage‐independent colony formation assay, which is a key characteristic of the transformed cell phenotype.^20^ Our data showed that treatment with paroxetine decreased colony formation by about 50% starting at a concentration of 5 μmol/L in both the cell lines. (Figure 2A). Consistent with the cell viability data, HT29 cells showed greater decrease in colony number at 10 μmol/L of paroxetine than the HCT116 cells.

These effects were further confirmed in the spheroid formation assay performed using a 3D cell culture model. Cell aggregation and spheroid formation were assessed by culturing HCT116 and HT29 cells in the presence of various concentrations of paroxetine for 1 week, and then spheroid formation was monitored. Captured images of spheroid formation at various time‐points are shown in Figure 2B. The cells gathered in the centre and formed round‐shaped clusters 1 hour after seeding, but treatment with 20 μmol/L paroxetine significantly reduced the cluster forming ability of HT29 cells (Figure 2B). In all clusters, cellular spheroid formation was initiated by spontaneous aggregation from 2 days after seeding, whereas HT29 cells treated with 20 μmol/L paroxetine still remained in the cluster stage (Figure 2B, 2 days). At day 6, spheroids continued to grow in vehicle‐treated HCT116 and HT29 cells, but treatment with paroxetine at concentrations from 5 μmol/L in both the cell lines inhibited growth after spheroid formation (Figure 2C). Moreover, spheroid formation was fully inhibited in 20 μmol/L paroxetine‐treated HCT116 cells and 10 μmol/L paroxetine‐treated HT29 cells. These results indicate that paroxetine has inhibitory effects on spheroid formation and growth of HCT116 and HT29 cells.

### 3.3 Paroxetine induces apoptosis in HCT116 and HT29 cells

**Figure 3:**
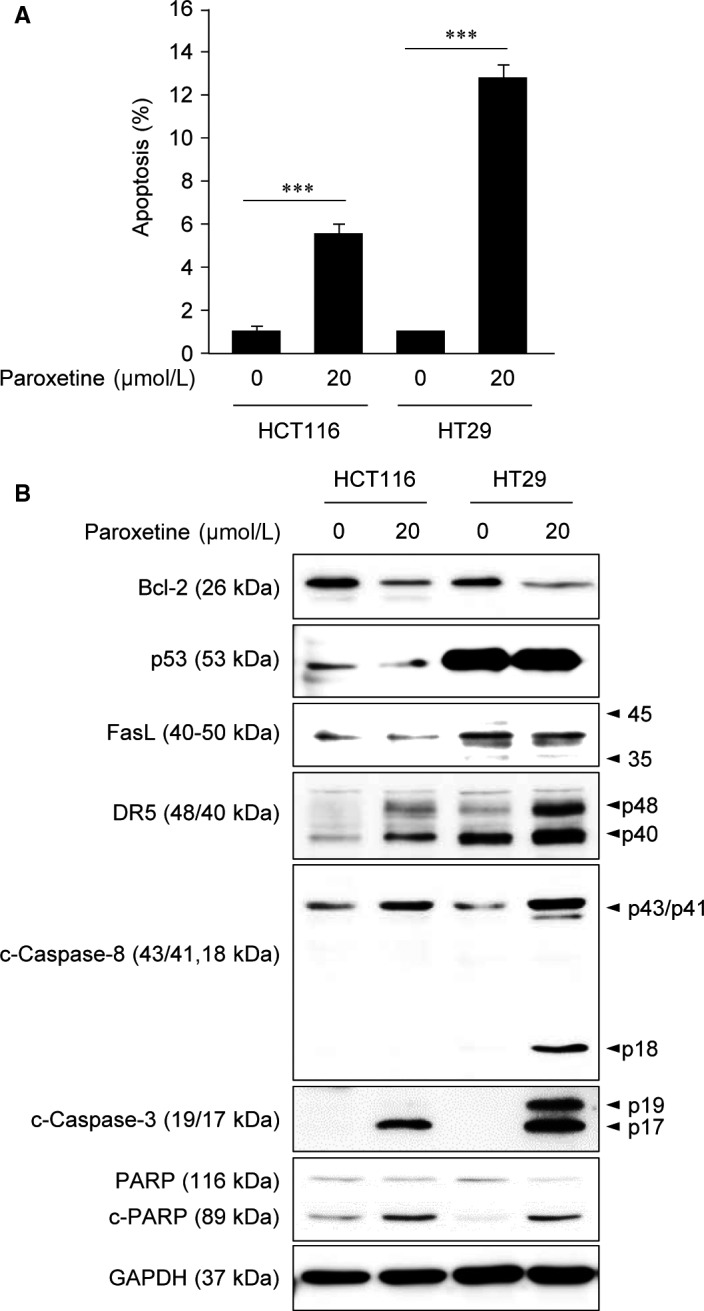
Effects of paroxetine on cell apoptosis in HCT116 and HT29 cells. A, Flow cytometry analysis after treatment with 10 μmol/L paroxetine for 24 h. Apoptotic cell death in HCT116 and HT29 cells was measured using the Annexin V‐FITC apoptosis detection kit. Results are shown as mean ± SD. Statistical significance was determined using a Student's *t* test (***P <* *0.001). B, Western blot analysis of pro‐ and anti‐apoptotic proteins after treatment with paroxetine for 24 h. GAPDH was used as a loading control

Next, apoptosis induction of paroxetine was assessed by detecting Annexin V‐positive cells by using flow cytometry. Treatment with 20 μmol/L paroxetine induced 5% and 13% apoptosis in HCT116 and HT29 cells, respectively. (Figure 3A). Western blot analysis showed a remarkable increase in activated caspase‐3 and PARP in both cell lines after treatment with 20 μmol/L paroxetine. Furthermore, the level of the anti‐apoptotic protein Bcl‐2 was decreased after treatment with 20 μmol/L paroxetine (Figure 3B), implying that paroxetine trigger intrinsic pathway of apoptosis. Interestingly, the activated form of caspase‐8 was increased after treatment with 20 μmol/L paroxetine in HCT116 and HT29 cells (Figure 3B). In addition, the elevated basal level of p53 was detected in HT29 cells (mutated p53), not in HCT116 cells (wild‐type p53) as reported earlier.^21^ However, p53 levels were not changed by paroxetine in both cell lines (Figure 3B), implying that paroxetine may play a role in p53‐independent manner. Similar to p53, we confirmed that the expression of Fas ligand (L) was not changed in the paroxetine‐treated cells compared with the nontreated cells (Figure 3B). However, expression levels of death receptor (DR)5, which could recruit the initiator caspase 8 and result in the cell death signalling cascade,^22^, ^23^ were up‐regulated in both cell lines after treatment with paroxetine (Figure 3B). Although DR5 was up‐regulated in both cells, more dramatic change was observed in HT29 cells after treatment with paroxetine, which suggesting the involvement of extrinsic apoptotic signalling pathway. Together, these results demonstrated that paroxetine induced apoptosis through intrinsic pathway by inhibition of Bcl‐2 and through extrinsic pathway by activation of DR5/caspase 8 signalling.

### 3.4 Paroxetine alters the phosphorylation of RTKs and downstream signalling in HCT116 and HT29 cells

**Figure 4:**
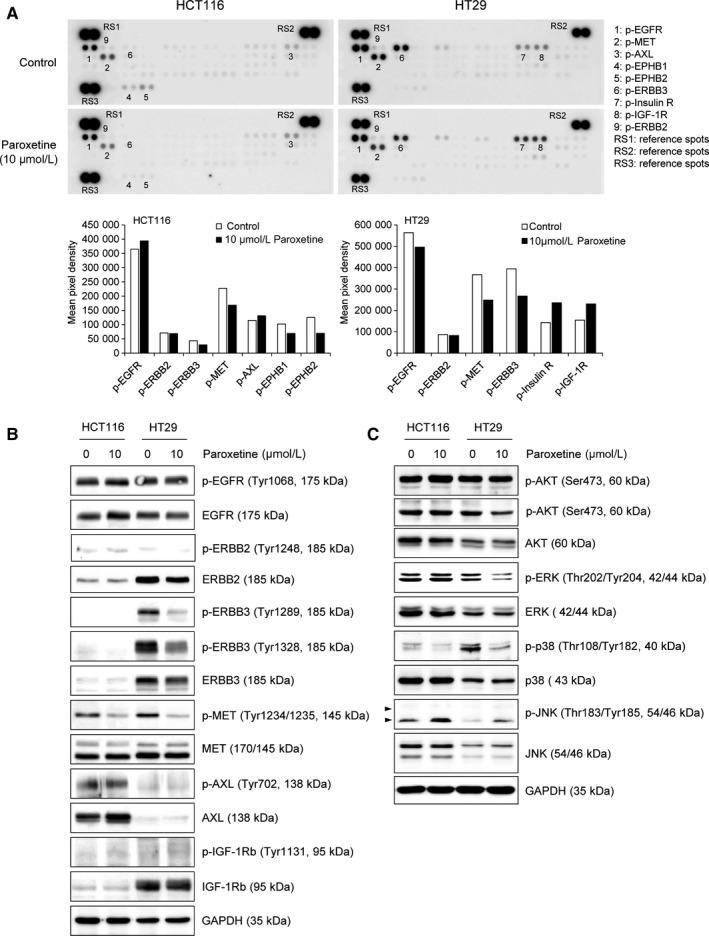
Paroxetine induces molecular alterations in HCT116 and HT29 cells. A, Phospho‐RTK analysis of HCT116 and HT29 cells. Cells were treated with 10 μM paroxetine for 24 h, and cell lysates were assayed using a human phospho‐RTK array kit. Phosphorylation levels were measured using Image J software and normalized to reference spots (R1, R2, and R3). The measured results (pixel density) are shown in a bar graph. B, Expression of several RTKs and phospho‐RTKs. Whole cell lysates were assayed using western blot analysis by using antibodies against total or phosphorylated RTKs. C, Expression of downstream molecules of RTKs. Whole cell lysates were assayed using western blot analysis by using antibodies against total or phosphorylated AKT, ERK, p38, and JNK. GAPDH was used as a loading control

Next, the underlying mechanism by which paroxetine exhibits different anticancer activity in HCT116 and HT29 cells was investigated. For this, a human phospho‐RTK array was performed, and phosphorylation levels of different RTKs after treatment with paroxetine were compared. Impressively, the levels of the phosphorylated forms of MET, EPHB1, and EPHB2 were decreased in HCT116 cells, whereas those of phosphorylated‐EGFR and ‐AXL remained unchanged after treatment with 10 μmol/L paroxetine (Figure 4A). Conversely, the phosphorylated levels of EGFR, MET, and ERBB3 were decreased in HT29 cells, whereas those of phosphorylated‐insulin R and IGF‐1R were increased by treatment with 10 μmol/L paroxetine (Figure 4A). Notably, phosphorylated‐MET was commonly detected and markedly decreased after treatment with paroxetine in both HCT116 and HT29 cells. However, the phosphorylation levels of ERBB2 in both HCT116 and HT29 cell lines were low and there was no significant difference in phosphorylated ERBB2 between the paroxetine treated cells and control cells (Figure 4A).

The expression and phosphorylation levels of RTKs were then confirmed by performing western blot analysis. Data showed that EGFR and MET were detected in both the cell lines. ERBB3 and IGF‐1Rb were only expressed in HT29 cells, whereas AXL was only expressed in HCT116 cells (Figure 4B). Consistent with the findings of phospho‐RTK array data, phosphorylated‐MET was decreased after treatment with 10 μmol/L paroxetine, whereas the phosphorylation levels of EGFR and ERBB2 remained unchanged in both the cell lines, indicating that MET might be a common pathway targeted by paroxetine in both HCT116 and HT29 cells (Figure 4B). Moreover, the phosphorylation level of ERBB3 in HT29 cells was significantly inhibited, whereas alterations of the phosphorylation status of IGF‐1Rb in HT29 and AXL in HCT116 cells was not changed after treatment with 10 μmol/L paroxetine (Figure 4B). These results might explain why HT29 cells were more sensitive to paroxetine treatment than HCT116 cells.

AKT, ERK, p38, and JNK are important kinases in the RTK downstream signalling pathways. Therefore, the expression and phosphorylation levels of these proteins were investigated. The results revealed that, the phosphorylation level of p38 and JNK were commonly altered by treatment with paroxetine in both HCT116 and HT29 cells **(**Figure 4C). Furthermore, the level of phosphorylated‐AKT was slightly reduced and phosphorylated‐ERK was markedly decreased in 10 μmol/L paroxetine‐treated HT29 cells (Figure 4C). To identify the role of JNK in paroxetine‐elicited apoptosis, we co‐treated HCT116 and HT29 colorectal cancer cells with paroxetine and SP600125 (Sigma, S5567), a pharmacological inhibitor of JNK. The results revealed that the activation of JNK by paroxetine was eliminated by SP600125 and the active forms of caspase‐8 and ‐3 induced by paroxetine were almost diminished by JNK inhibitor in both cell lines (Figure S1). Taken together, these data suggest that paroxetine suppresses cancer progression via the inhibition of the common RTK pathway involving MET‐p38, and JNK in both the cell lines. In addition to the common pathway, the ERBB3‐ERK signalling pathway is also highlighted in HT29 cells, thereby providing a molecular mechanism by which paroxetine exhibits higher anticancer effect in HT29 cells.

### 3.5 Paroxetine inhibits tumour growth in a HT29‐xenograft model

**Figure 5:**
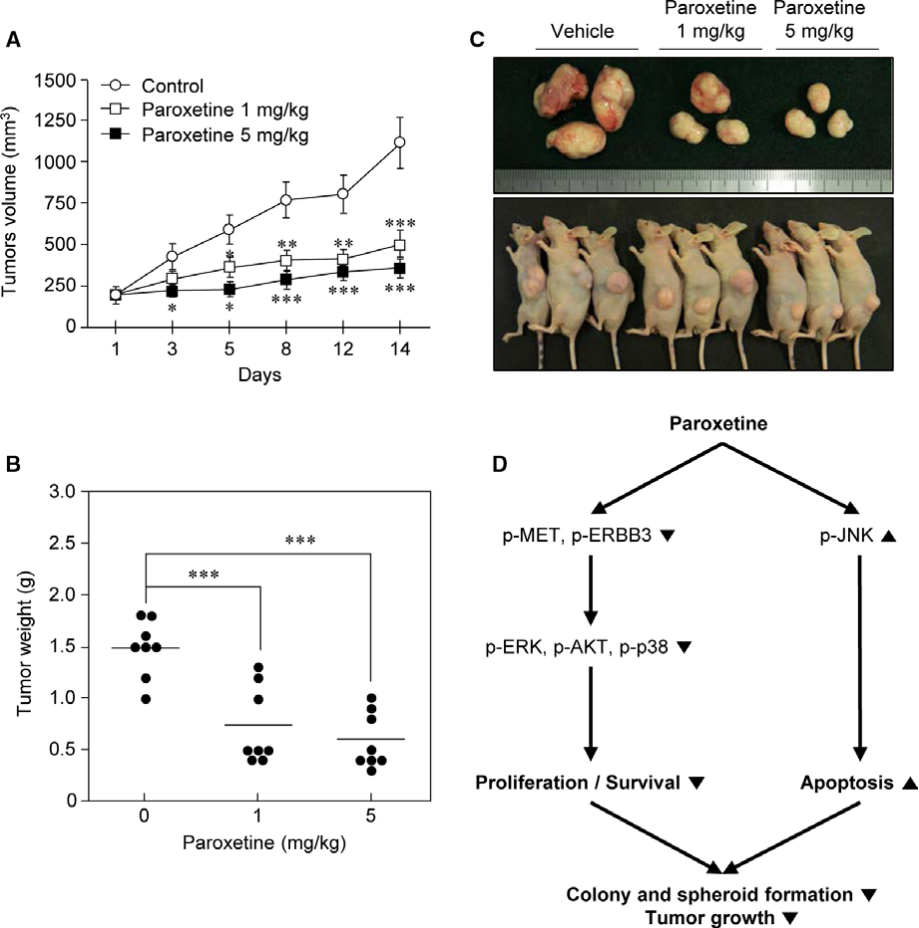
Paroxetine inhibits xenograft tumour growth of HT29 cells. HT29 cells were subcutaneously injected into athymic nude mice for the development of xenograft tumours. Vehicle or 1 or 5 mg/kg paroxetine was injected intraperitoneally three times per week for 2 wk. A, Tumour volume was calculated according to a standard formula: tumour volume (mm^3^) = (length × width × height × 0.5). Data are presented as mean ± SD (n* *=* *8). Statistical significance was determined using Student's *t* test (*P <* *0.05, **P <* *0.01, ***P <* *0.001). B, Tumour weight was recorded after excision on the day of the termination of the experiment. Data are presented as mean ± SD (n* *=* *8). ***P <* *0.001 when compared to the control. C, Tumour size was measured three times per week by using calipers. D, Simplified diagram of the anticancer mechanism of paroxetine in colon cancer cells

The direct anticancer activity of paroxetine in vivo was determined by subcutaneously transplanting HT29 cells into athymic nude mice. After treatment with 1 or 5 mg/kg paroxetine 3 times per week, remarkable inhibition of tumour growth was noted in HT29‐xenograft mice, resulting in a significantly lower tumour volume (Figure 5A) and weight (Figure 5B) than those in controls after 2 weeks of therapy. Representative photographs of the tumours that developed in mice are shown in Figure 5C.

## 4 DISCUSSION

Our study proposes a molecular mechanism whereby paroxetine restrains CRC cell growth and survival, leading to the inhibition of tumourigenesis in vivo. Paroxetine is able to inhibit the activity of RTKs, which are highly expressed and play an essential role in CRC development. Therefore, paroxetine induces the alteration of downstream signalling pathways, including suppression of AKT, ERK, and p38 and activation of JNK, resulting in the activation of caspases, which are the decisive regulators of apoptosis.

Recently, drug repositioning studies have revealed the anti‐cancer effect of paroxetine in various types of cancer cells. Serafeim et al reported that paroxetine induces the inhibition of DNA synthesis in biopsy‐like Burkitt lymphoma cells.^19^ Levkoviz et al showed that paroxetine caused the inhibition of cell growth and induction of apoptosis in rat C6 glioma cells and human SH‐SY5Y neuroblastoma cells.^10^ Chou et al showed that paroxetine decreases cell viability and induces apoptosis in human MG63 osteosarcoma cells.^16^ Gil‐Ad et al found that paroxetine induces the inhibition of cell growth in human HT29 and LS1034 colon adenocarcinoma cell lines.^15^ Kuwahara et al showed the apoptotic effect of paroxetine in human HepG2 hepatocellular carcinoma cells.^9^ Consistent with the findings of these studies, our findings revealed the capacity of paroxetine to cause cell growth inhibition, cell death induction, and anticancer effect in HT29‐xenografted mice.

Programmed cell deaths are vitally important processes for maintaining the morphological patterns and physiological tissue homeostasis during development.^24^ Apoptosis, a mode of cell death, is a crucial and common response to cytotoxic treatments. Activation of caspases and PARP are the central events underlying apoptosis.^24^ Apoptosis can be initiated through one of two pathways: intrinsic and extrinsic pathways. Activation of death receptors such as Fas or TNF‐α receptors by their ligands transmits death signals to the intracellular signalling pathways, resulting in the activation of caspase‐8. In turn, active caspase‐8 causes the cleavage of caspase‐3 and ‐7, leading to widespread cell death.^25^, ^26^ Alternatively, the intrinsic pathway can be activated by diverse non‐receptor‐mediated stimuli. These stimuli induce the activation of one or more members of the BH3‐only protein family against the anti‐apoptotic activity of B‐cell lymphoma‐2 (Bcl‐2) family members. This causes mitochondrial outer membrane permeabilization, followed by the release of pro‐apoptotic proteins from the intermembrane space of the mitochondria into the cytosol, thereby initiating the apoptosis program.^26^, ^27^, ^28^ Here, our study revealed that treatment with paroxetine induced up‐regulation of DR5 death receptor by tumour necrosis factor‐related apoptosis‐inducing ligand (TRAIL) independent manner, leading to the activation of caspase 8, followed by the activation of caspase‐3 (Figure 3B). As a result, extrinsic apoptotic events were triggered, causing cell death by paroxetine treatment. In addition, paroxetine treatment also induced the decreased expression of Bcl‐2 – an anti‐apoptotic protein (Figure 3B). Thereby, the apoptotic proteins such as caspase‐3 and PARP were activated, inducing intrinsic apoptotic program together with extrinsic events, resulting in the death of paroxetine treated HCT116 and HT29 cells.

In our study, the IC_50_ values for paroxetine in HT29 cells was lower than that in HCT116 cells, implying the differences in sensitivity depending on the type of tumour cells (Figure 1B, C). Moreover, at the same concentration of paroxetine, the apoptotic effect on HT29 cells was more than twice as that in HCT116 cells (Figure 3A). The different susceptibility of HCT116 and HT29 cell lines to paroxetine could be attributed to the differences in the response of these cell lines to paroxetine. Indeed, paroxetine‐induced apoptosis in HT29 cells appears to be associated with decreased activity of ERBB3, which might further inhibit ERK and accelerate cell death (Figure 4B, C).

The cellular biological processes, including cell proliferation, survival, and apoptosis, are intricately controlled by many signalling cascades. Among these cascades, RTKs are one of the important regulators of the signalling cascade involving cell differentiation, cell proliferation, and apoptosis.^29^ RTKs are generally activated by dimerization and conformational changes, followed by tyrosine phosphorylation after the binding to their growth factors.^30^ EGFR of the EGF receptor family or also called ERBB receptor family is the first RTK to be discovered in 1978.^31^ ERBB3 is another member of the EGFR family. Although ERBB3 has no intrinsic tyrosine kinase activity, it can transfer signals by interacting with other kinase active receptors such as EGFR, ERBB2, and ERBB4.^32^ Phosphorylation at the tyrosine residues of these proteins activates the downstream pathways such as Ras/MAPK, PLCγ1/PKC, PI3K/Akt, and STAT, promoting cell proliferation. Another major member of RTKs that plays an essential role in the regulation of cell survival is IGF‐IR. Because of the essential roles of these RTKs in cell growth, their inhibition is one of the key targets of cancer therapy. Therefore, we focused on RTKs, which are thought to play an important role in cell proliferation, survival, and cell death, by performing phosphor‐RTK array and western blot analysis. Both these assays showed a remarkable decrease in the activation of MET in both HCT116 and HT29 cell lines (Figure 4A, B). As expected, a marked difference in activated RTK pattern was observed (Figure 4A). We found that activated EGFR, MET, AXL, EPHB1, and EPHB2 were basally detected in HCT116 cells (Figure 4A, left) and phosphorylated EGFR, MET, ERBB3, Insulin R, and IGF‐1R were basally detected in HT29 cells (Figure 4A, right). MET is known to be abnormally activated in many cancers, and its activation has been reported to contribute to tumour growth, proliferation, and survival.^33^, ^34^ These patterns were further confirmed by western blot analysis. Interestingly, phosphorylated MET decreased by paroxetine treatment in both the cell lines, suggesting that MET could be a common target for paroxetine (Figure 4B). Conversely, phosphorylated ERBB3 was highly detected exclusively in HT29 cells and significantly decreased after treatment with paroxetine (Figure 4B). ERBB3 is also a protein known to be overexpressed and mutated in cancer cells. Many studies have shown that ERBB3 contributes to tumourigenesis, proliferation, and cell survival in cancer.^35^ Downstream signalling of these RTKs revealed that the phosphorylation of ERK, p38, and AKT were markedly down‐regulated. Therefore, the reduction in activated MET and ERBB3 causing the down‐regulation of active AKT, ERK, and p38 by paroxetine might lead to the inhibition of cell growth and survival (Figure 5D). In addition, inhibition of the anti‐apoptotic effect of MET and ERBB3 might be one causes of paroxetine‐induced cell death. Furthermore, the in vivo study results confirmed the in vitro data that paroxetine downgraded tumour growth in xenografted mice. Our findings suggest that paroxetine did not target a single tyrosine kinase receptor, but multiple RTKs simultaneously, advocating paroxetine as a promising anticancer agent in CRC therapy.

## CONFLICT OF INTEREST

The authors declare no conflicts of interest.
