# Supplementary figures and images for: Anticancer activity of paroxetine in human colon cancer cells: Involvement of MET and ERBB3
Source: J Cell Mol Med. 2018 Nov 13;23(2):1106–15. doi: 10.1111/jcmm.14011 (PMC6349215; doi:10.1111/jcmm.14011)

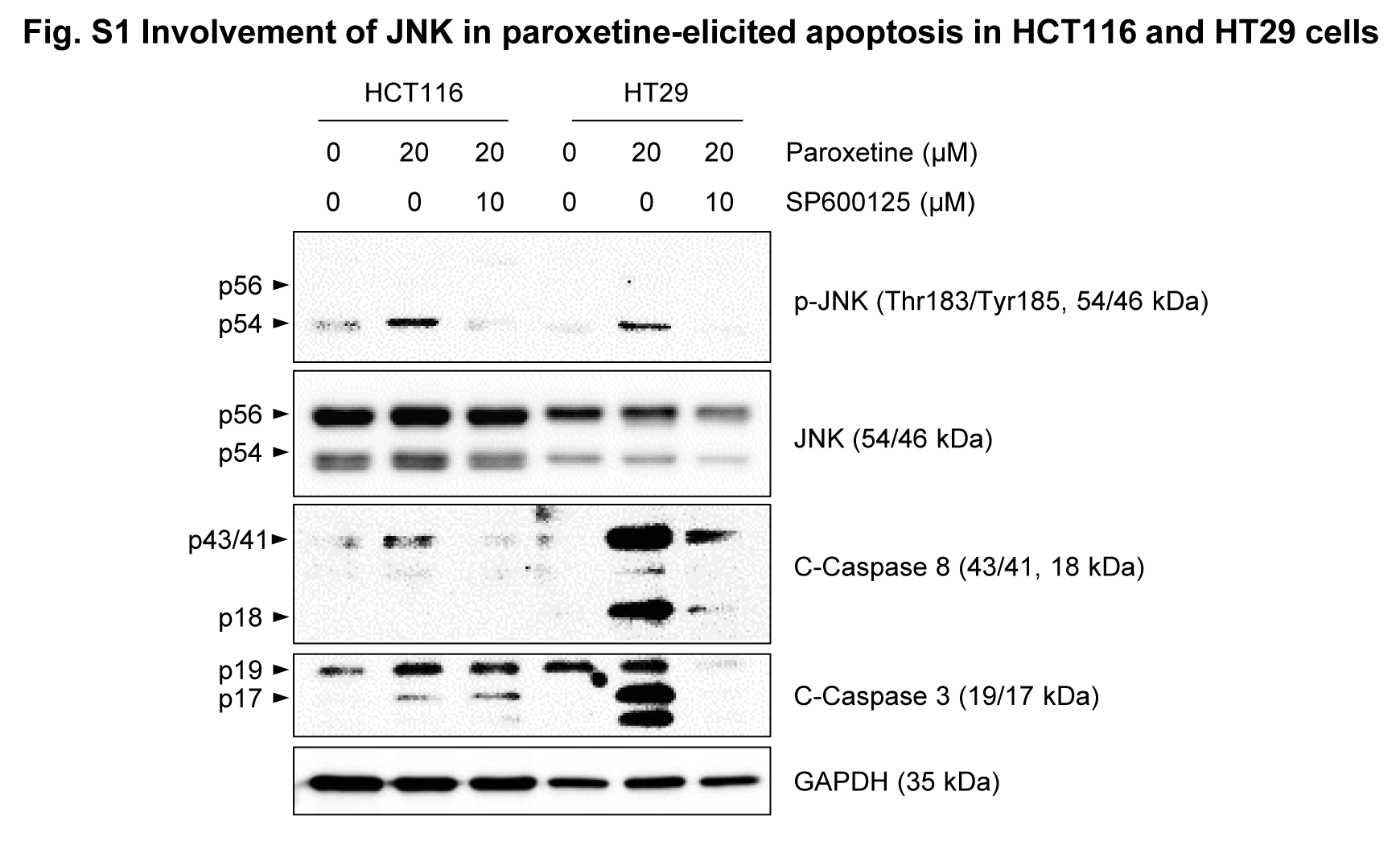

Supplement: Supplementary file 1 [file JCMM-23-1106-s001.tif]
